# Chinese Herbal Medicine for the Treatment of Prehypertension

**DOI:** 10.1155/2013/493521

**Published:** 2013-07-01

**Authors:** Jie Wang, Bo Feng, Xiaochen Yang, Wei Liu, Xingjiang Xiong

**Affiliations:** Department of Cardiology, Guang'anmen Hospital, China Academy of Chinese Medical Sciences, Beixiange 5, Xicheng District, Beijing 100053, China

## Abstract

*Objectives*. To assess the current clinical evidence of Chinese herbal medicine (CHM) for prehypertension. *Search Strategy*. Electronic databases were searched until May, 2013. *Inclusion Criteria*. We included randomized clinical trials testing CHM against life style intervention and no treatment, or combined with life style intervention against life style intervention. *Data Extraction and Analyses*. Study selection, data extraction, quality assessment, and data analyses were conducted according to Cochrane standards. *Results*. Five trials were included. Methodological quality of the trials was evaluated as generally low. Only 1 trial reported allocation sequence. No trial reported the allocation concealment, double blinding, placebo control, presample size estimation, intention to treat analysis, and drop-out. All the included trials were not multicenter and large scale. Although meta-analysis showed that CHM is superior to either life style intervention group or no treatment group in decreasing blood pressure, we are unable to draw a definite conclusion on the effect of CHM due to the poor research methods used in the reviewed trials. The safety of CHM is still uncertain. *Conclusions*. There is no evidence to show that CHM is effective and safe for prehypertension due to serious methodological flaw of the reviewed trials. Rigorously designed trials are warranted to confirm these results.

## 1. Introduction

In 2003, the Seventh Joint National Committee on the Prevention, Detection, Evaluation, and Treatment of Hypertension (JNC-7) introduced a new category based on blood pressure (BP) level, called “prehypertension,” to designate individuals whose systolic blood pressure (SBP) levels are in the range of 120 to 139 mmHg and diastolic blood pressure (DBP) between 80 and 89 mmHg [1, 2]. As compared to normal BP, prehypertension is a precursor of clinical hypertension, which is associated with increased long-term risk and cardiovascular morbidity and mortality [3–5]. It is demonstrated by many researches that the rate of progression is determined mostly by age and resting blood pressure but may also be attenuated by increased fitness [6]. In recent observational studies in population-based samples with mean ages from 40 to 80 years, the risk of cardiovascular diseases (CVDs) increased progressively from levels as low as 115/75 mmHg upward with a doubling of the incidence of both coronary heart disease and stroke for every 20/10 mmHg increment of BP [7]. That is to say, the higher the BP, the greater the chance of heart attack, heart failure, stroke, and kidney diseases.

Currently, the prevention and management of prehypertension are the major public health challenges. According to JNC-7, the objective of defining this classification was to draw required clinical and public healthy attention and health promoting lifestyle modifications at an even earlier stage to prevent the progressive rise in BP and cardiovascular disease. Therefore, the primary goal of managing prehypertension is to lower BP into the normal range, prevent a rise in BP with age, and prevent BP-related CVDs events. Management consists of nonpharmacological interventions (including appropriate dietary pattern, weight loss, reduced sodium intake, regular physical activity, and moderation of alcohol intake) and pharmacological interventions [8, 9]. Perhaps if prehypertension was eliminated, almost half of all heart attacks could be prevented [10–12]. 

With the increasing enhancement of people's awareness of self-care, drugs with natural medicinal plants are increasingly favored by people all over the world for their unique advantages in preventing and curing diseases, rehabilitation, and health care [13–15]. In East Asia (especially China), a certain proportion of the population with prehypertension or hypertension has turned to complementary and alternative medicine (CAM), including Chinese herbal medicine (CHM) [16–19], for lowering BP and improving its related symptoms [20–22]. CHM, which is the most important component of traditional Chinese medicine (TCM), has a 3000-year-old history with unique theories for concepts of etiology and systems of diagnosis and treatment [23]. The knowledge about how to use them has been passed down through generations by word of mouth and ancient pharmacopoeias [24, 25]. Modern science has devoted considerable research to characterizing the efficacy and mechanisms of action of CHM. It is demonstrated that CHM has a vast array of bioactivities and is used in the management of various CVDs [26–29]. Recently, increasing number of clinical trials and systematic reviews (SRs) have been conducted, showing that CHM appears to be effective in lowering BP smoothly [30–36].

Until now, a number of clinical trials of CHM for prehypertension have been reported with positive findings [37–43]. However, there is no critically appraised evidence such as SRs or meta-analysis to assess clinical efficacy and safety of CHM for prehypertension. The paper aims to evaluate the current clinical evidence of CHM for prehypertension in randomized trials. To our knowledge, this is the first systematic English review on CHM for prehypertension.

## 2. Methods

### 2.1. Database and Search Strategies

The literature searches were conducted in the following 7 electronic databases: Cochrane Library (January, 2013), EMBASE (1980–2013), PubMed (1959–2013), Chinese National Knowledge Infrastructure (CNKI) (1980–2013), Chinese Scientific Journal Database (VIP) (1989–2013), Chinese Biomedical Literature Database (CBM) (1978–2013), and Wanfang data (1998–2013). The reference list of retrieved papers was also searched. Databases in Chinese were searched to retrieve the maximum possible number of trials of CHM for prehypertension because CHM is mainly practiced and studied in China. All of those searches ended on 1 May, 2013. Ongoing registered clinical trials were searched in the website of Chinese clinical trial registry (http://www.chictr.org/) and international clinical trial registry by US national institutes of health (http://clinicaltrials.gov/). The following search terms were used individually or combined: “prehypertension,” ‘‘high-normal blood pressure,” “blood pressure,” “Chinese herbal medicine,” “herb,” “herbal medicine,” “Chinese herb,” “traditional Chinese medicine,” “clinical trial,” and “randomized controlled trial.” 

### 2.2. Inclusion Criteria

All the parallel randomized controlled trials (RCTs) of CHM used alone versus nonpharmacologic interventions, no treatment, and conventional western medicine were included. RCTs combined CHM with nonpharmacological interventions or conventional western medicine versus nonpharmacological interventions or conventional western medicine were included as well. There were no restrictions on population characteristics, language, and publication type. The main outcome measure was blood pressure. Duplicated publications reporting the same groups of participants were excluded.

### 2.3. Data Extraction and Quality Assessment

Two authors conducted the literature searching (X. J. Xiong, B. Feng), study selection (X. J. Xiong, X. C. Yang), and data extraction (X. J. Xiong, W. Liu) independently. The extracted data included authors, title of study, year of publication, study size, age and sex of the participants, study characteristics, diagnosis standard, details of methodological information, name and component of Chinese herbs, treatment process, details of the intervention and control, outcomes, and adverse effects for each study. Disagreement was resolved by discussion and reached consensus through a third party (J. Wang).

The methodological quality of trials was assessed independently using criteria from the Cochrane Handbook for Systematic Review of Interventions, Version 5.1.0 (X. J. Xiong, B. Feng) [44]. The items included the following 7 aspects: random sequence generation (selection bias), allocation concealment (selection bias), blinding of participants and personnel (performance bias), blinding of outcome assessment (detection bias), incomplete outcome data (attrition bias), selective reporting (reporting bias), and other bias. The quality of all the included trials was categorized to low/unclear/high risk of bias (“Yes” for a low risk of bias, “No” for a high risk of bias, “Unclear” otherwise). Then, trials were categorized into three levels: low risk of bias (all the items were in low risk of bias), high risk of bias (at least one item was in high risk of bias), unclear risk of bias (at least one item was in unclear risk of bias).

### 2.4. Data Synthesis

RevMan 5.1 software provided by the Cochrane Collaboration was used for data analyses. Dichotomous data were presented as risk ratio (RR) and continuous outcomes as mean difference (MD), both with 95% confidence interval (CI). Heterogeneity was recognized significant when *I* ^2^ ≥ 50%. Fixed effects model was used if there is no significant heterogeneity of the data; random effects model was used if significant heterogeneity existed (50% < *I* ^2^ < 85%). Publication bias would be explored by funnel plot analysis if sufficient studies were found.

## 3. Result

### 3.1. Description of Included Trials

**Figure 1:**
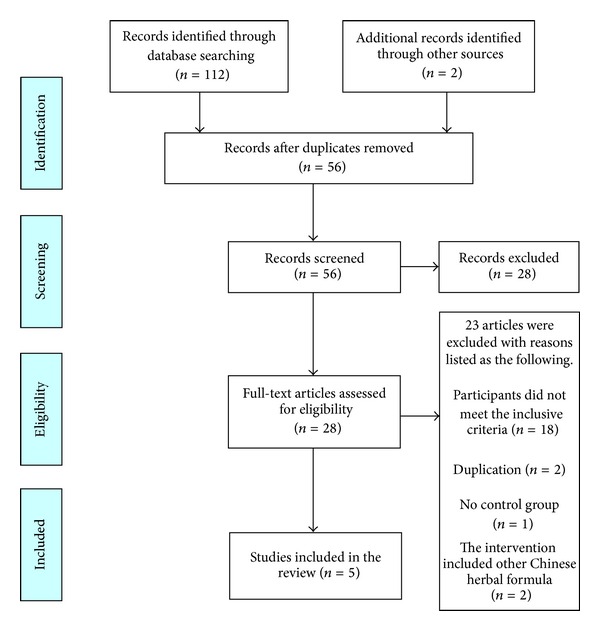
PRISMA 2009 flow diagram.

As shown in Figure 1, the flow diagram depicted the search process and study selection. After primary searches from the above 7 electronic databases, 112 articles were retrieved. Fifty-Six articles were screened after 58 duplicates were removed. After reading the titles and abstracts, 28 articles were excluded. Full texts of 28 articles were retrieved, and 23 articles were excluded with reasons listed as below: participants did not meet the inclusive criteria (*n* = 18), duplication (*n* = 2), no control group (*n* = 1), and the intervention included other Chinese herbal formula (*n* = 2). Finally, 5 RCTs [45–49] were included. All of them were published in Chinese. The characteristics of included trials were listed in Table 1.

Four hundred and thirty prehypertensive patients were included. There was a wide variation in the age of subjects (23–75 years). Five trials specified the same diagnostic criteria of prehypertension, that is, Seventh Report of the Joint National Committee on Prevention, Detection, Evaluation, and Treatment of High Blood Pressure (JNC-7) [45–49]. Three trials have reported TCM diagnostic criteria according to Guidelines of Clinical Research of New Drugs of Traditional Chinese Medicine (GCRNDTCM) [45, 46, 48]. One trial reported prehypertensive patients with liver fire syndrome (LFS) [45]; 1 trial reported prehypertensive patients with abundant phlegm-dampness syndrome (PDS) [46]; 1 trial reported prehypertensive patients with LFS, PDS, yin deficiency with yang hyperactivity syndrome (YDYHS), and deficiency of both yin and yang syndrome (DYYS), respectively [48]. The other 2 trials have not reported any TCM diagnostic criteria at all [47, 49].

Interventions included CHM used alone or combined with life style intervention. The controls included life style intervention alone or no treatment. Three trials investigated CHM combined with life style intervention versus life style intervention [45, 46, 48]; 1 trial investigated CHM used alone versus no treatment [47]; 1 trial investigated CHM used alone versus life style intervention [49].

Total treatment duration ranged from 4 to 12 weeks. The variable prescriptions were presented in Table 1. The different compositions of CHM were presented in Table 2. All of the 5 included trials used BP as the outcome measure. Adverse effect was described in details.

### 3.2. Methodological Quality of Included Trials

Methodological quality of the majority of the included trials was assessed to be low according to the predefined quality assessment criteria (as shown in Table 3). Randomized allocation of participants was mentioned in all trials; however, only 2 trials reported the methods for sequence generation including random number table [45, 46]. No detailed information was provided in the other 3 trials to judge whether or not it was conducted properly [47–49]. Allocation concealment, blinding of participants and personnel, and blinding of outcome assessment were not mentioned in all included trials. None of the trials have reported drop-out or withdraw. A pretrial estimation of sample size was not reported in all the trials. One trial mentioned the follow-up with 6 months [46], while the other 4 trials did not mention it at all [45, 47–49]. We have also tried to contact the author by telephone, fax, and email for further detailed information about the trials; however, no information has been provided to date.

### 3.3. Effect of the Interventions

#### 3.3.1. Chinese Herb Medicine Plus Life Style Intervention versus Life Style Intervention

**Figure 2:**
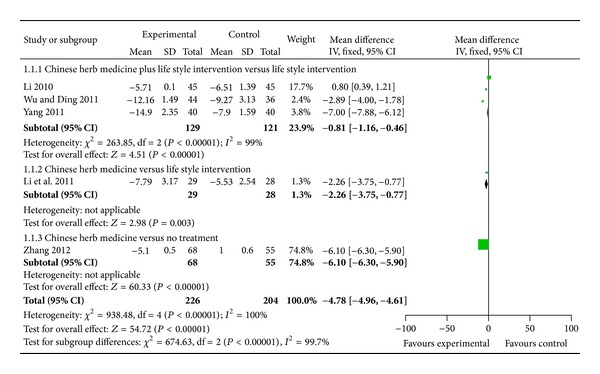
Analyses of systolic blood pressure (SBP).

Three trials reported the effect of CHM plus life style intervention versus life style intervention [45, 46, 48]. When it comes to systolic blood pressure (SBP), 2 trials demonstrated better effect favoring CHM: Banxia baizhu tianma decoction mildly lowered SBP than life style intervention treatment [46]; tiao ping kang pill significantly lowered SBP than life style intervention treatment [48]. Meta-analysis showed beneficial effect on the combination group as compared to the life style intervention group (WMD: −0.81 [−1.16, −0.46]; *P* < 0.00001) (as shown in Figure 2).

**Figure 3:**
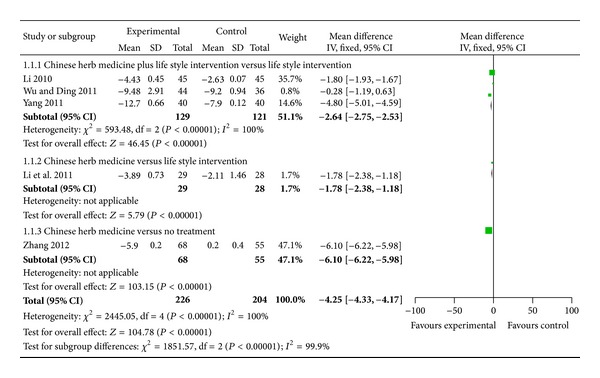
Analyses of diastolic blood pressure (DBP).

When it comes to diastolic blood pressure (DBP), 2 trials demonstrated better effect favoring CHM: Chinese herb medicine combined with life style intervention mildly lowered DBP than life style intervention [45]; the combination of tiao ping kang pill and life style intervention significantly lowered DBP than life style intervention [48]. Meta-analysis showed beneficial effect on the combination group as compared to the life style intervention group (WMD: −2.64 [−2.75, −2.53]; *P* < 0.00001) (as shown in Figure 3).

#### 3.3.2. Chinese Herb Medicine versus Life Style Intervention

One trial reported CHM used alone versus life style intervention [49]. When it comes to SBP, meta-analysis showed that there is beneficial effect on the blood pressure-lipid lowering decoction group as compared to the life style intervention group (WMD: −2.66 [−3.75, −0.77]; *P* = 0.003) (as shown in Figure 2).

When it comes to DBP, meta-analysis showed that there is beneficial effect on the blood pressure-lipid lowering decoction group as compared to the life style intervention group (WMD: −1.78 [−2.38, −1.18]; *P* < 0.00001) (as shown in Figure 3).

#### 3.3.3. Chinese Herb Medicine versus No Treatment

1 trial reported CHM used alone versus no treatment [47]. When it comes to SBP, meta-analysis showed that there is significant beneficial effect on the replenishing qi and strengthening spleen decoction group as compared to no treatment group (WMD: −6.10 [−6.30, −5.90]; *P* < 0.00001) (as shown in Figure 2).

When it comes to DBP, meta-analysis showed there is significant beneficial effect on the blood pressure-lipid lowering decoction group as compared to no treatment group (WMD: −6.10 [−6.22, −5.98]; *P* < 0.00001) (as shown in Figure 3).

### 3.4. Publication Bias

The number of trials was too small to conduct any sufficient additional analysis of publication bias.

### 3.5. Adverse Effect

The outcome of adverse events (AEs) was reported in only 1 trial [49]. In the trial of Zhang 2012, no AEs were found in life style intervention group. AEs in CHM group included abdominal distension and diarrhea. And they were not serious. The other 4 trials have not been mentioned at all [45–48].

## 4. Discussion

Evidence suggests that individuals with BP close to the traditional threshold for the diagnosis of hypertension have a high likelihood of progression to BP meeting the definition of hypertension over the ensuing 5 years [50]. Therefore, the control of BP in a timely manner is of great significance for promoting cardiovascular health in prehypertensive patients. It is worth noting that the goal of therapy happens to coincide with ancient preventive medicine in TCM, that is, ‘‘the earlier the better for treating who and what are not yet ill [Sic]” in ‘‘Huang di nei jing” and “Nan jing” classics [29, 51, 52]. Due to the health-enhancing qualities of CHM, it has been dispensed and used in China for many years. Current researches demonstrated that CHM possess the advantage of whole body regulation in many ways for many targets. Recently, the continued study of the antihypertensive mechanisms of CHM for lowering BP has made great progress with regard to the etiology and pathogenesis of this disease. As an adjunctive treatment to antihypertensive drugs, CHM is a popular natural herbal product for prehypertension. However, the role of CHM for prehypertension is still unclear. This study aims to assess the current clinical evidence of CHM for prehypertension.

This systematic review included 5 randomized trials and a total of 430 participants. In this review, several CHMs demonstrated potential positive effect for prehypertension on either SBP or DBP. As compared to life style intervention group, positive results in SBP (WMD: −0.81 [−1.16, −0.46]; *P* < 0.00001) and DBP (WMD: −2.64 [−2.75, −2.53]; *P* < 0.00001) were found about Chinese herb medicine plus life style intervention group, indicating that SBP and DBP could be decreased by 0.81 mmHg and 2.64 mmHg, respectively, after the combination therapy. As compared to life style intervention group, positive results in SBP (WMD: −2.66 [−3.75, −0.77]; *P* = 0.003) and DBP (WMD: −1.78 [−2.38, −1.18]; *P* < 0.00001) were found about Chinese herb medicine group, indicating that SBP and DBP could be decreased by 2.66 mmHg and 1.78 mmHg, respectively, after CHM therapy. As compared to no treatment group, positive results in SBP (WMD: −6.10 [−6.30, −5.90]; *P* < 0.00001) and DBP (WMD: −6.10 [−6.22, −5.98]; *P* < 0.00001) were found about Chinese herb medicine group, indicating that SBP and DBP could be decreased by 6.10 mmHg and 6.10 mmHg, respectively, after CHM therapy.

However, due to the poor methodological qualities, lack of placebo controlled trial and repeated test, small reduction in BP, and significant heterogeneity of included trials, available data are not adequate to draw a definite conclusion on the therapeutic effect and safety of CHM for prehypertension, although meta-analysis showed that CHM is superior to either life style intervention group or no treatment group in decreasing blood pressure. The following reasons might contribute to the inconclusive results.

Firstly, the quality of all the included RCTs is generally low, which were in accordance with previous studies [53, 54]. All the 5 trials included in this paper had risk of bias in terms of design, reporting, and methodology. They provided only inadequate reporting of study design, allocation sequence, allocation concealment, blinding, intention to treat analysis, and drop outs account in the majority of trials. Only 1 RCT stated randomization procedure with table of random number. However, most of them just mentioned that ‘‘prehypertensive patients were randomized into two groups” without detailed information. Thus, insufficient information has greatly restricted us to judge whether the randomization was conducted properly, which might lead to potential selection bias. None of the included RCTs reported the allocation concealment. Therefore, we could not rule out the possibility that some of these claimed RCTs are not real RCTs. Additionally, no RCTs claimed blinding of participants and personnel and blinding of outcome assessment, which directly led to performance bias and detection bias. Maybe there was difficulty in conducting the blinding of participants and personnel; however, none of the trials used blinding of outcome assessment, as the data analyzed by a third party could be conducted much easier. What is more, no RCTs used placebo control in the 5 included trials. Perhaps certain features associated with Chinese herbs such as aroma, color, and appearance did limit the clinical usage of placebo. However, it might exaggerate the effect of CHM due to the lacking of placebo, which was prone to generate significant systemic errors in the assessment of outcomes. All the included RCTs have not reported presample size estimation and drop-out. And most of the trials have not reported intention to treat analysis in details. All the included trials were not multicenter and large scale. As known to all, if poorly designed, the conclusions would show larger differences between well-designed and poorly designed trials. Therefore, due to serious research methodological flaw in the included trials, the credibility of research findings in our review might be greatly reduced. And the reported beneficial effect from CHM for prehypertension cannot be taken as confirmative conclusion.

Secondly, there was a lack of knowledge about the significance of reporting AEs in the RCTs. AE is a critical issue in CAM which has raised more and more concern world widely [55–57]. In China, there is a general view that it seems to be safe to use CHM for various diseases [58, 59]. In our review, most of the trials did not report the adverse effect of CHM except one [49]. Even for the trials that reported AEs by Zhang 2012, the report was very brief with insufficient information. Therefore, a definite conclusion about the safety of CHM cannot be made.

Thirdly, heterogeneity is worthy of being paid attention to. A total of 27 different Chinese herbs were investigated in the 5 RCTs. Great heterogeneity existed in these CHMs of this review. As a result, it is impossible to conduct any meaningful meta-analysis for a specific Chinese herb or difficult to undertake subgroup analyses to explore the specific factors that may have an impact on the effects of the treatment regimen. 

Fourthly, a syndrome is a unique concept in TCM theory [24, 60]. In the practice of TCM, CHM should be consistent with the type of syndrome differentiation. Therefore, TCM syndrome becomes the key issue both for RCT and clinical practice, which is also known as treatment based on individualized pattern and is thought to be the advantage of TCM [61, 62]. However, in this systematic review, only 2 trials provide detailed information on patients' syndrome differentiation [45, 46]. Two trials did not report any TCM diagnostic criteria [47, 49]. One trial used tiao ping kang pill as the intervention; however, 4 TCM syndromes were reported, which have brought great confusion for further analysis [48]. We cannot exclude the possibility that the patients were not treated according to syndrome differentiation.

In conclusion, there is no convincing evidence of CHM for prehypertension due to poor methodological quality of included trials. To ensure evidence-based clinical practice, further rigorously designed placebo-controlled, randomized trials are warranted to confirm the results. The following methodological issues should be addressed carefully: (a) appropriate methods used in generating allocation sequence and allocation concealment; (b) double blinding (blinding of participants and personnel and blinding of outcome assessment) with the rational use of placebo; (c) strictly reporting withdrawal/dropout and the usage of intention to treat analysis; and (d) comprehensively reporting trials according to the CONSORT Statement [63]. We hope that with increasing publication of new high-qualified RCTs, more convincing clinical evidence would show whether or not CHM is safe and effective for prehypertension.

## Figures and Tables

**Table 1:** Characteristics and methodological quality of included studies.

Study ID	Sample	Diagnosis standard	Intervention	Control	Course (week)	Outcome measure
Yang 2011 [45]	90	JNC-7; GCRNDTCM	CHM (800 mL/d^#^) + control	Life style intervention	4	BP
Li 2010 [46]	80	JNC-7; GCRNDTCM	BBTD (400 mL/d^#^) + control	Life style intervention	4	BP
Li et al. 2011 [47]	123	JNC-7; TCM diagnostic criteria (unclear)	RQSPD (200 mL/d^#^)	No treatment	4	BP
Wu and Ding 2011 [48]	80	JNC-7; GCRNDTCM	TPKP (1.2 g tid) + control	Life style intervention	8	BP
Zhang 2012 [49]	57	JNC-7; TCM diagnostic criteria (unclear)	BPLLD (1 bag bid)	Life style intervention	12	BP

Note: CHM: Chinese herb medicine; BBTD: Banxia baizhu tianma decoction; RQSPD: replenishing qi and strengthening spleen decoction; TPKP: tiao ping kang pill; BPLLD: blood pressure-lipid lowering decoction.

**Table 2:** Composition of formula.

Study ID	Formula	Composition of formula
Yang 2011 [45]	CHM	Mulberry leaves 10 g, chrysanthemum 10 g, hawthorn 10 g, rose 10 g.
Li 2010 [46]	BBTD	Pinellia ternata 10 g, atractylodes macrocephala 15 g, gastrodia elata 15 g, tangerine peel 10 g, poria cocos 15 g, glycyrrhiza 5 g, ginger 10 g, and red jujube 10 g.
Li et al. 2011 [47]	RQSPD	Lanceolata 20 g, kudzu root 20 g, astragalus 15 g, poria cocos 15 g, atractylodes macrocephala 15 g, rhizoma atractylodes 15 g, coix lachryma-jobi 15 g, trichosanthin 15 g, pinellia ternata 10 g, and tangerine peel 10 g.
Wu and Ding 2011 [48]	TPKP	Ligustrum lucidum, epimedium, leonurus japonicus, and so forth.
Zhang 2012 [49]	BPLLD	Chrysanthemum, cassia seed, sophora flower, hawthorn, lotus leaf, alisma orientalis, and green tea.

Note: CHM: Chinese herb medicine; BBTD: Banxia baizhu tianma decoction; RQSPD: replenishing qi and strengthening spleen decoction; TPKP: tiao ping kang pill; BPLLD: blood pressure-lipid lowering decoction.

**Table 3:** Quality assessment of included randomized controlled trials.

Included trials	Random sequence generation	Allocation concealment	Blinding of participants and personnel	Blinding of outcome assessment	Incomplete outcome data	Selective reporting	Other sources of bias	Risk of bias
Yang 2011 [45]	Table of random number	Unclear	Unclear	Unclear	Yes	No	Unclear	Unclear
Li 2010 [46]	Table of random number	Unclear	Unclear	Unclear	Yes	No	Unclear	Unclear
Li et al. 2011 [47]	Unclear	Unclear	Unclear	Unclear	Yes	No	Unclear	High
Wu and Ding 2011 [48]	Unclear	Unclear	Unclear	Unclear	Yes	No	Unclear	High
Zhang 2012 [49]	Unclear	Unclear	Unclear	Unclear	Yes	No	Unclear	High
